# Supplementary material for: A Selective Adenylyl Cyclase 1 Inhibitor Relieves Pain Without Causing Tolerance
Source: Front Pharmacol. 2022 Jul 11;13:935588. doi: 10.3389/fphar.2022.935588 (PMC9310748; doi:10.3389/fphar.2022.935588)
Supplement: Supplementary file 1 [file DataSheet1.PDF]

Supplementary Material

A selective adenylyl cyclase 1 inhibitor relieves pain without causing tolerance

Gianna Giacoletti, Tatum Price, Lucas V.B Hoelz, Abdulwhab Shremo Msdi, Samantha Cossin, Katerina Vazquez-Falto, Tácio V. Amorin Fernandes, Núbia Boechat, Adwoa Nornoo, Tarsis F. Brust

Correspondence to: tbrust@atyrpharma.com

1.1 Supplementary Figures

Plot statistics

Residues in most favoured regions [A,B,L]	302	88.0%
Residues in additional allowed regions [a,b,l,p]	29	8.5%
Residues in generously allowed regions [~a,~b,~l,~p]	7	2.0%
Residues in disallowed regions	5	1.5%
	----	------
Number of non-glycine and non-proline residues	343	100.0%
Number of end-residues (excl. Gly and Pro)	7	
Number of glycine residues (shown as triangles)	32	
Number of proline residues	9	

Total number of residues	391	

Based on an analysis of 118 structures of resolution of at least 2.0 Angstroms and R-factor no greater than 20%, a good quality model would be expected to have over 90% in the most favoured regions.

Supplementary Figure 1. Ramachandran plot statistics analysis of the adenylate cyclase 1 (AC1) model made on the SAVES server using PROCHECK.

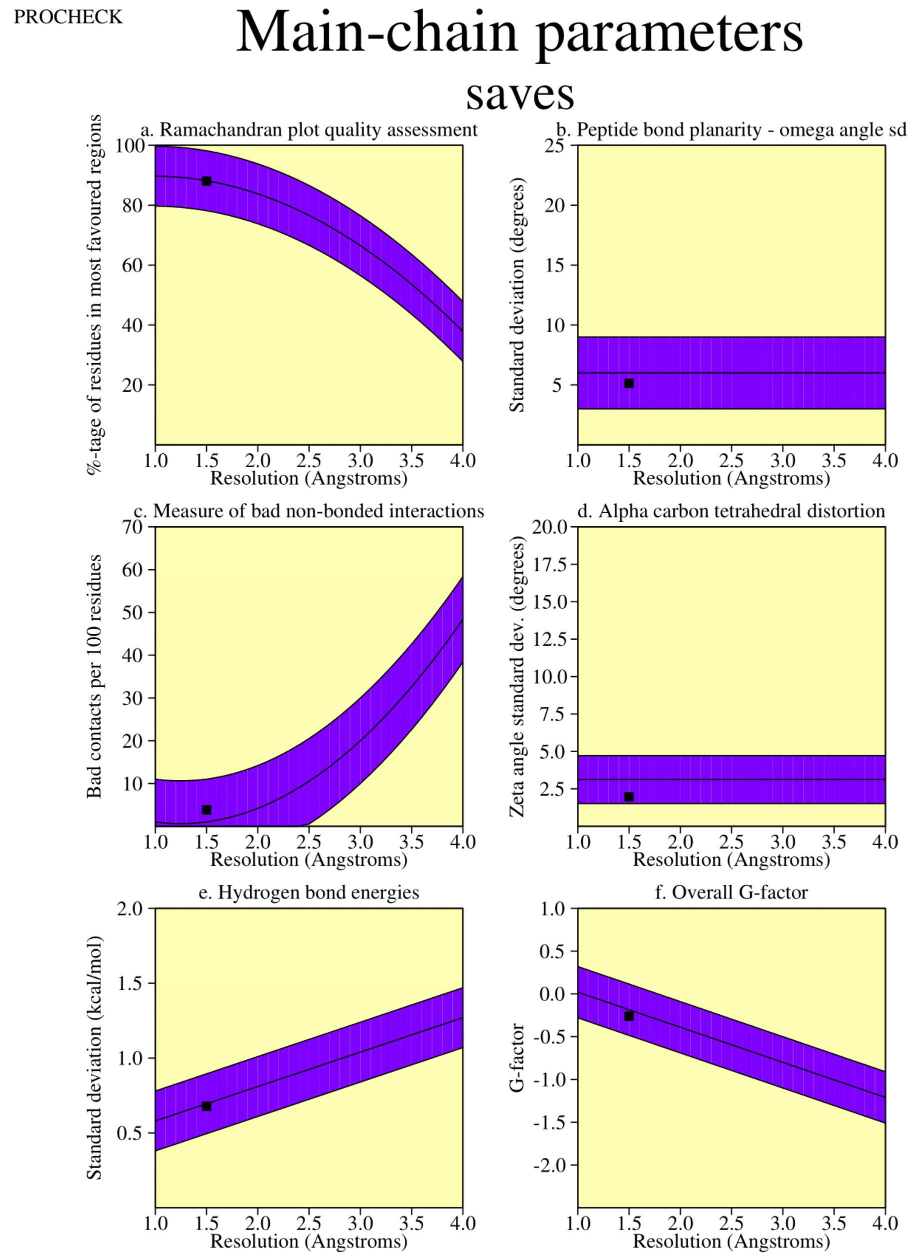

Plot statistics

Stereochemical parameter	No. of data pts	Parameter value	Comparison values Typical value	Band width	No. of band widths from mean	
a. %-tage residues in A, B, L	343	88.0	88.2	10.0	-0.0	Inside
b. Omega angle st dev	385	5.1	6.0	3.0	-0.3	Inside
c. Bad contacts / 100 residues	15	3.8	1.0	10.0	0.3	Inside
d. Zeta angle st dev	355	2.0	3.1	1.6	-0.7	Inside
e. H-bond energy st dev	256	0.7	0.7	0.2	-0.1	Inside
f. Overall G-factor	391	-0.3	-0.2	0.3	-0.3	Inside

Supplementary Figure 2. Main-chain stereochemical parameters statistical analysis of the AC1 model made on the SAVES server using PROCHECK.

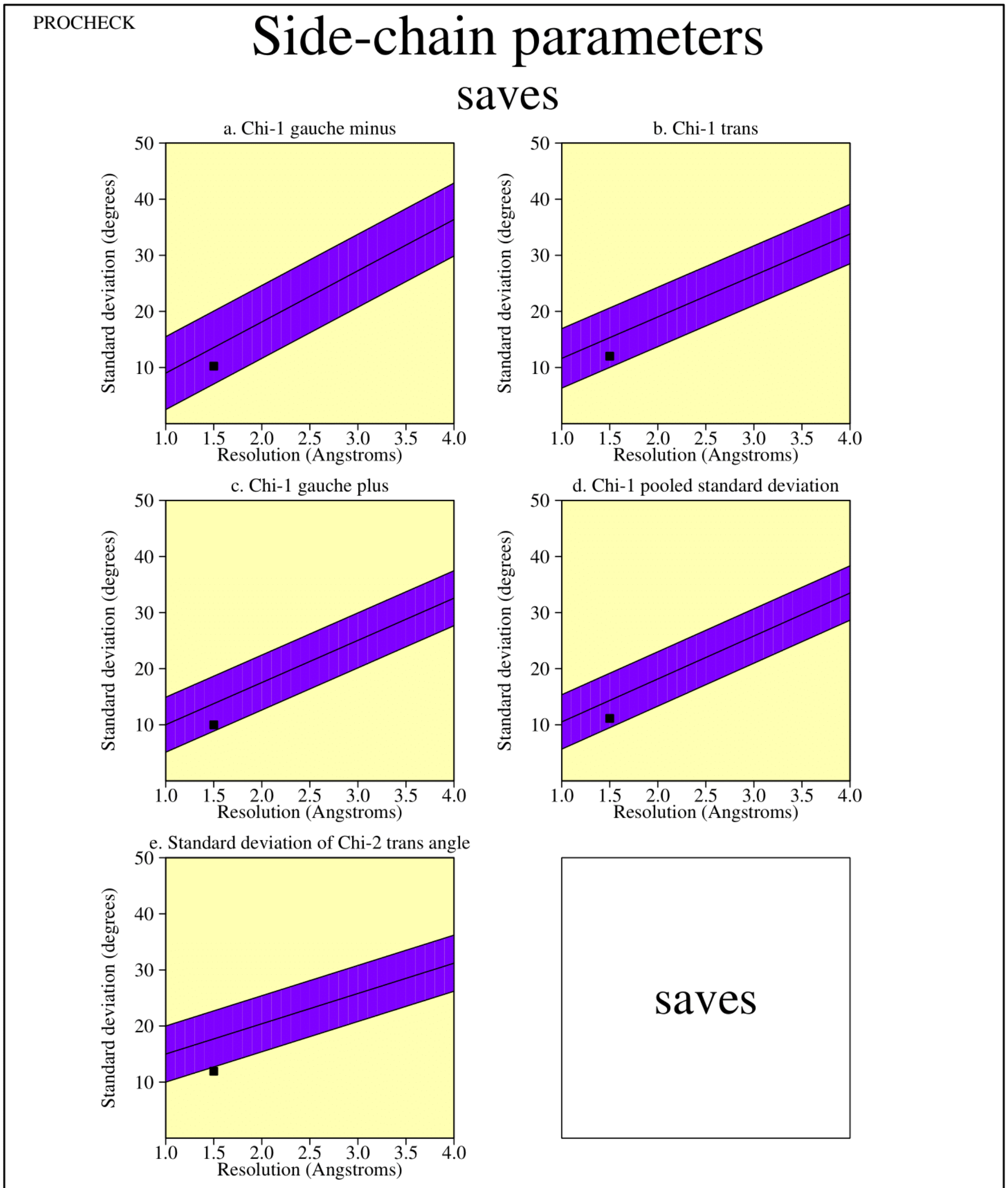

Plot statistics

Stereochemical parameter	No. of data pts	Parameter value	Comparison values Typical value	Band width	No. of band widths from mean	
a. Chi-1 gauche minus st dev	67	10.2	13.6	6.5	-0.5	Inside
b. Chi-1 trans st dev	127	12.0	15.3	5.3	-0.6	Inside
c. Chi-1 gauche plus st dev	130	10.0	13.8	4.9	-0.8	Inside
d. Chi-1 pooled st dev	324	11.1	14.3	4.8	-0.7	Inside
e. Chi-2 trans st dev	64	11.9	17.7	5.0	-1.2	BETTER

Supplementary Figure 3. Side-chain stereochemical parameters statistical analysis of the AC1 model made on the SAVES server using PROCHECK.

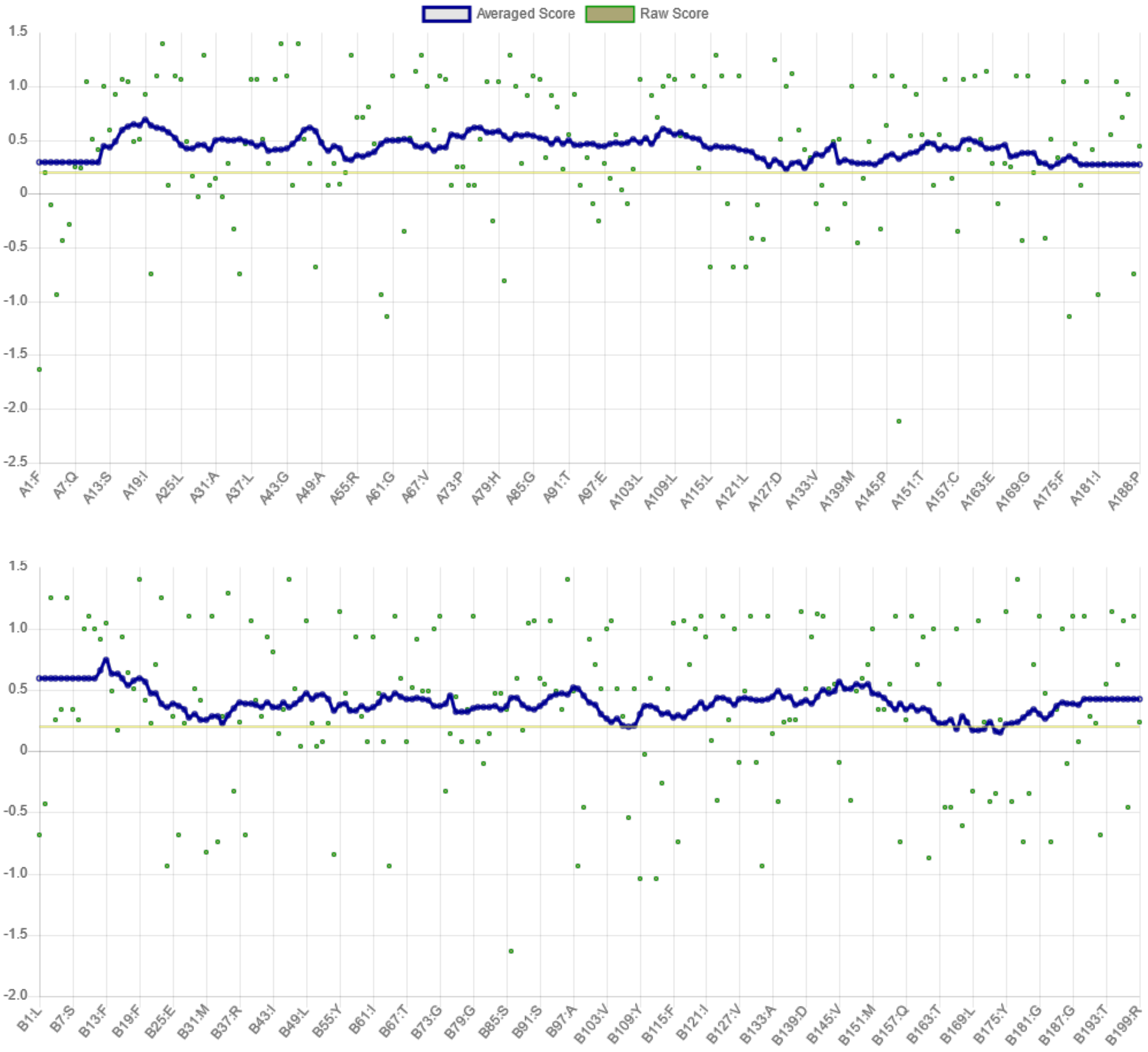

Supplementary Figure 4. Three-dimensional profiles analysis of the AC1 model made on the SAVES server using VERIFY 3D for chain C1a and C2a, respectively. 98.45% of the residues have averaged 3D-1D score >= 0.2.

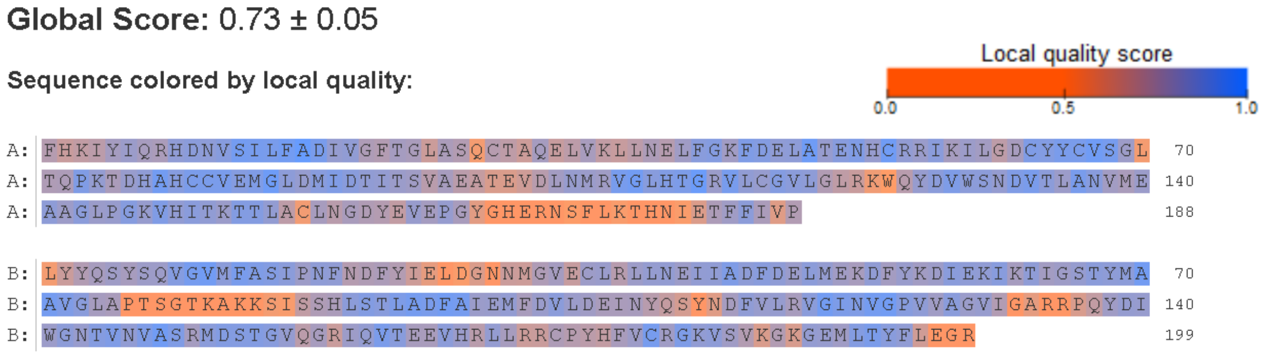

Supplementary Figure 5. QMEANDisCo analysis of the AC1 model made on the SWISS-PROT server. QMEANDisCo scored regions of the model from 0 to 1, or poor to good, based on statistical potentials of mean force and agreement terms with consensus-based distance constraints.

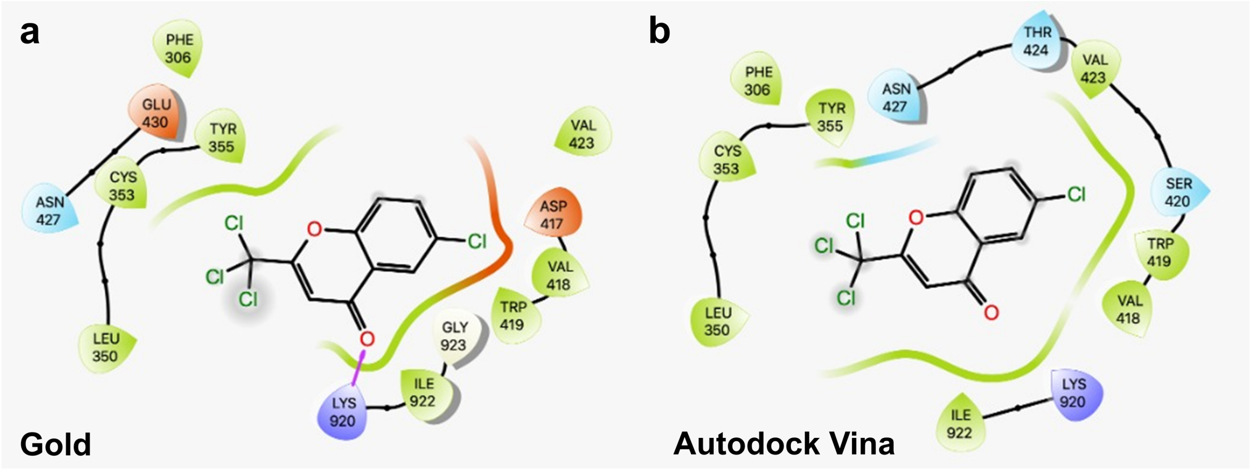

Supplementary Figure 6. 2D representation of the ST034307 poses, using Gold (A) and Autodock Vina (B) programs, showing the hydrogen bond (purple arrows) and steric interactions with the AC1 model.
